# Assessing influenza activity variations in the Asian region during the pre- and post-pandemic period (2017–2023)

**DOI:** 10.1371/journal.pone.0323465

**Published:** 2025-06-04

**Authors:** Nadia Nisar, Nazish Badar, Inayah Safdar

**Affiliations:** Public Health Laboratories Division National Institute of Health, Islamabad, Pakistan; Universiti Brunei Darussalam, BRUNEI DARUSSALAM

## Abstract

**Background:**

The year 2021 witnessed a decline in seasonal influenza cases across Southeast Asia and the broader Asian region. However, a sudden surge in influenza cases during 2022–2023 necessitates comprehensive exploration and analysis to inform future prediction models.

**Objective:**

Our study aims to evaluate the disease burden of influenza in Asian countries post-COVID-19, while comparing seasonal variations to the pre-pandemic influenza patterns.

**Methods:**

We conducted an extensive analysis of data spanning from January 2017 to September 2023 across ten Asian countries, categorizing them into three WHO regions. Data was sourced from the WHO Flunet system, falling under the purview of the WHO Global Influenza Program.

**Findings and conclusion:**

In conclusion, influenza epidemiology during the inter-pandemic period is characterized by seasonality influenced by factors such as population contact patterns, virus survival, and host immunity. The year 2020 witnessed a global decrease in influenza circulation due to widespread lockdowns and travel restrictions. However, a resurgence was observed in late 2021, notably with out-of-season activity in the Southern Hemisphere. Our analysis based on reviews indicates a probable significant increase in influenza cases in the upcoming seasons. To address this, the implementation of influenza vaccination programs and the promotion of vaccination for both children and adults are essential measures to alleviate the dual burden of influenza in the post-COVID era.

## Introduction

Influenza is a pervasive respiratory infection that afflicts populations worldwide. According to the World Health Organization (WHO) report from January 2023, seasonal influenza is responsible for an annual toll ranging from 290,000–650,000 deaths globally, with the highest mortality rate observed among individuals aged 65 and above [1]. Regions such as Southeast Asia and Africa bear the heaviest burden, exhibiting mortality rates ranging from 3.5 to 9.2 per 100,000 individuals, whereas the United States reports a comparatively lower average mortality rate of 1.4 per 100,000 individuals [2].

The emergence of COVID-19 has heightened concerns about increased vulnerability to Influenza viruses, potentially leading to more substantial influenza outbreaks worldwide. Consequently, proactive measures to predict seasonal influenza trends within WHO regions have become essential. In this study, our objective is to forecast variations in influenza activity and provide insights into future seasonal trends across Asian countries, particularly following the lifting of COVID-19 restrictions in many nations [3].

Post-pandemic, in July 2022, influenza type A experienced a resurgence in Southeast Asian countries, notably Malaysia, the Philippines, and Thailand, reminiscent of pre-pandemic activity levels. Although influenza activity in both the southern and northern hemispheres, in 2022, remained lower than pre-COVID levels, but comparatively the influenza activity was still higher than pre COVID activity. By February 2023, countries in East Asia, including Malaysia, Thailand, and Singapore, witnessed an upsurge in influenza activity [4]. Global influenza activity remained elevated as of January 2023, with Pakistan and other South Asian countries reporting a resurgence in influenza A(H1N1) pdm09 (Pandemic Disease Mexico 2009) [3]. Furthermore, the co-occurrence of Severe Acute Respiratory Syndrome Coronavirus 2 (SARS-CoV-2) and influenza was observed in September 2022, leading clinicians to prioritize testing for both influenza A and B, alongside SARS-CoV-2, for high-priority patients [5].

The phenomenon of reduced influenza transmission during COVID-19 can be attributed to viral interference between SARS-CoV-2 and the influenza virus within affected individuals [6]. A concept originally described by the research group of Voroshilova in the 1960s [7]. Data from the Center for Disease Control (CDC) supports this notion, revealing a decline in influenza rates during the COVID-19 pandemic period, extending into spring 2021 [8]. Notably, Brazil reported only 1.62% of cases as influenza-positive in 2020. However, CDC data for late 2021 to early 2022 shows peaks in influenza positivity, specifically in weeks 2, 3, and 14 of 2022 [9].

In temperate Asian zones, such as Pakistan and India, influenza experiences tertiary seasons from October to February each year. During peak seasons, swift data evaluation of influenza cases is imperative for Public Health Institutes. Unfortunately, low- to medium-income countries often lack community-based surveillance (CBS) or vaccination systems for high-risk groups. The co-detection of influenza and COVID-19 suggests that flu immunization may have been affected, although it has still played a role in controlling influenza incidence to a certain extent. From October 2022 to April 2023, the CDC estimated that influenza caused 19,000–58,000 deaths in the United States [10]. Therefore, a global perspective on influenza spread is essential to determine influenza subtypes amid the backdrop of COVID-19. Surveillance capacity within Asian countries predicts seasonal influenza resurgence in densely populated areas such as India, China, and Southeast Asia [11].

The disruptive impact of COVID-19 on post-pandemic influenza activity has made it challenging to interpret influenza data during the years 2021–2022. Furthermore, influenza vaccination efforts in countries like the United States have been influenced by COVID vaccination campaigns [12]. These factors introduce uncertainty regarding future influenza epidemics, as reduced virus exposure and affected immunity come into play [13]. The seasonal trend of influenza has been dysregulated since the conclusion of the COVID-19 pandemic, posing critical questions about future strategies for influenza outbreak control.

This study encompasses three WHO regions: SEAR (South-East Asia Region), WPR (Western Pacific Region), and EMR (Eastern Mediterranean Region). Within SEAR, countries such as India, Bangladesh, Nepal, Sri Lanka, Thailand, Indonesia, and Myanmar, with a combined population of 687,909,327, play a pivotal role. Pakistan, with a population of 241,381,224, and Malaysia and the Philippines [14], with populations of 33.9 million and 113.9 million respectively, contribute significantly to the regional landscape.

The effect of COVID-19 on influenza transmission in the Southern and Northern hemispheres, particularly within temperate Asian regions, remains an ongoing mystery. Despite reduced reporting of influenza data post-COVID-19, middle Asian countries continue to experience resurgences, particularly involving the H3N2 strain [15]. Therefore, mitigating the risk of future public health infections during influenza outbreaks necessitates the collection of data during the COVID-19 pandemic period. The primary objective of our cross-sectional review is to evaluate the changes in influenza activity and its circulation throughout the pre-pandemic, pandemic, and post-pandemic periods. This understanding will inform the development of preventive programs for upcoming influenza seasons, supported by continuous monitoring of prevailing influenza outbreaks. Unlike previous studies that primarily focused on pre-COVID influenza spread within individual countries, our analysis encompasses multiple Southern and South-East Asian countries within WHO regions [16,17].

## Methods

### Data source

In conducting this review-based study, we carefully selected ten countries situated in Southern and Southeast Asia to ensure a comprehensive representation of the region. These countries encompass Pakistan, India, Bangladesh, Nepal, Sri Lanka, Malaysia, Thailand, Philippines, Myanmar, and Indonesia. The study is structured as a cross-sectional analysis, spanning both the pre-pandemic period (2017–2019) and the post-pandemic period (2020–2023).

The data utilized in our analysis originates from FluNet reports, which provide detailed information regarding the specimens received, specimens tested, and the percentage of positive cases over specified time intervals. The World Health Organization (WHO) Global Influenza Programme serves as the primary platform for the collection and analysis of this influenza surveillance data. This information is disseminated through FluNet and FluID by the Global Influenza Surveillance and Response System (GISRS) in collaboration with national epidemiological institutions. Notably, this data collection persisted even in the presence of concurrent reporting on COVID-19 cases.

Our study focused on gathering epidemiological data from each of the selected countries, specifically in terms of the number of specimens received, the specimens subjected to testing, and the count of influenza-positive specimens. Additionally, we systematically collected data pertaining to the timing and intensity of influenza activity through FluID influenza surveillance outputs, facilitating comparisons with previous years’ surveillance data. Flu Informed Decisions (FluID) served as the source for obtaining data concerning influenza and severe acute respiratory syndrome (SARS) positivity rates, which were subsequently submitted to FLUMART WHO [18].

The data sourced from the six WHO regions, namely the African Region (AFR), Region of the Americas (AMR), Eastern Mediterranean Region (EMR), European Region (EUR), South-East Asia Region (SEAR), and Western Pacific Region (WPR), is updated on a weekly basis. These reports encompass various influenza subtypes, including A[H1], A[H1N1]pdm09, A[H3N2], A[H5], A[H7N9], and B subtypes. We included all available surveillance systems in our study, comprising sentinel, non-sentinel, and those with undefined surveillance methodologies.

It is important to note that each country contributed data covering different annual ranges. The analysis were carried out on the influenza activity of ten Asian countries during the period from 2017 to 2019, as updated on FluNet. In order to provide a comprehensive overview, we incorporated non-sentinel data from countries that did not participate in sentinel surveillance. Furthermore, the results include information about peak influenza activity observed during the years 2017–2023. Ultimately, our analysis drew upon influenza data from these ten countries spanning a total of 347 weeks for comprehensive and rigorous examination.

### Case definition criteria

#### Influenza like illnesses.

WHO defines influenza like illnesses (ILI); an acute respiratory infection is a history of fever which is ≥ 38 C° and cough; with onset within the last 10 days.

#### Severe acute respiratory infection.

WHO defines severe acute respiratory infection (SARI): an acute respiratory infection is history of fever or measured fever of ≥ 38 C° and cough; with onset within the last 10 days and requires hospitalization [19].

### Statistical analysis

#### Normality test.

The assumption of normality for the data was assessed using the Shapiro-Wilk test, which yielded a W statistic of 0.898 and a p-value of 0.037. Since the *p*-value is below the threshold of 0.05, the null hypothesis of normality was rejected, indicating that the data significantly deviate from a normal distribution. Consequently, a non-parametric test, the Wilcoxon test, was chosen to analyze the data. Wilcoxon test was used for the independent and unpaired samples for pre and post pandemic data.

#### Wilcoxon test results.

**Null hypothesis (H_**0**_):** There is no significant difference in the Value distribution between the **Pre-COVID** and **Post-COVID** periods.

**Alternative hypothesis (H_**1**_):** There is a significant difference in the Value distribution between the **Pre-COVID** and **Post-COVID** periods.

The Wilcoxon rank sum exact test was conducted to compare “Pre-COVID” and “Post-COVID” values. The results are as follows:

W statistic: 16

*p*-value: 0.008931

Conclusion: Since the p-value is below 0.05, we reject the null hypothesis and conclude that there is a significant difference in values between the Pre-COVID and Post-COVID periods. The alternative hypothesis suggests that there is a difference between the two groups, and the *p*-value provides evidence in support of that.

**Fig 1:**
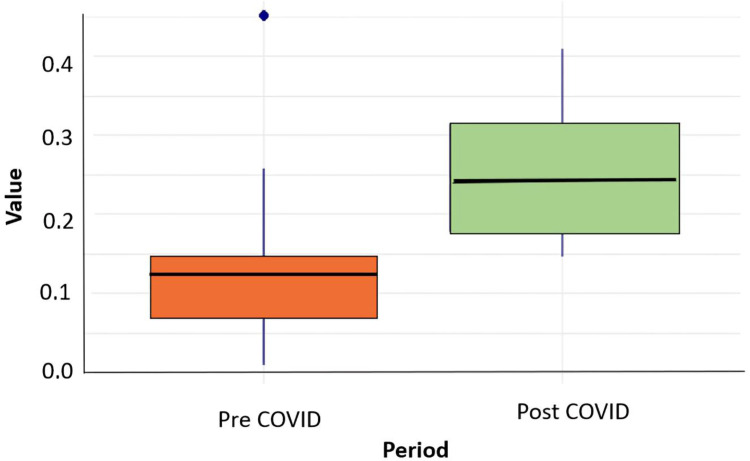
Boxplot rate of influenza positivity value by period.

The boxplot (Fig 1) illustrates that the median values are higher in the **“Post-COVID” period** compared to the **“Pre-COVID” period**, indicating an increase in the measured metric after the onset of the pandemic. Greater variability is observed in the “Post-COVID” group, as shown by the wider interquartile range, while the “Pre-COVID” data exhibits a tighter spread along with the presence of an outlier. These visual trends align with the Wilcoxon test results (W = 16, p = 0.008931), which confirm a statistically significant change in values between the two periods. This likely reflects the pandemic’s impact on the measured metric.

**Sample effect size (Cohen’s d) estimates:**

Table 1 gives Cohen’s d values for positive influenza cases and number of specimens tested for pre and post pandemic period. The small effect size (Cohen’s d = 0.21) suggests that the difference in influenza-positive cases before and after COVID-19 was not very large compared to overall variability in data. However, the total number of tests conducted after the pandemic was much higher (Cohen’s d = -0.56), likely due to more frequent testing during COVID 19 pandemic and overlapping symptoms between COVID 19 and influenza can be a factor. Despite this rise in testing, the number of positive cases did not increase as significantly. Similarly, boxplot analysis also showed higher median values and greater variability in spread of data during post pandemic period, while the Wilcoxon test (W = 16, p = 0.008931) confirms a statistically significant difference between the two periods.

**Table 1:** Effect Size Analysis of Influenza Testing and Specimen Detection before and after the COVID-19 Pandemic.

Characteristics	Mean [SD]	Effect Size, Cohen’s d
Pre pandemic positive influenza cases	4093.5 [3,868.94]	d = 0.21
Post pandemic positive influenza cases	3309.9 [3,517.95]	
Specimen tested Pre pandemic	18616.7 [21,753.86]	d = -0.56
Specimen tested post pandemic	42239.9 [54,731.69] [54,731.69]	

#### Confidence interval calculation.

The analysis of the data from the pre- and post-COVID periods included the estimation of 95% confidence intervals (CIs) for both periods using bootstrap resampling with 1,000 replicates via the boot.ci function in R. For the “Pre-COVID” period, the CI was (0.1716, 0.3465), while for the “Post-COVID” period, the CI was (0.067, 0.196). These intervals were calculated using the percentile method on the original data scale.

To assess the difference between the two periods, the Shapiro-Wilk test was applied, revealing a non-normal distribution of the data (W = 0.898, p = 0.037). As a result, a non-parametric Wilcoxon rank sum test was used, which showed a statistically significant difference between the “Pre-COVID” and “Post-COVID” periods (W = 16, p = 0.008931). The boxplot analysis further illustrated that the “Pre-COVID” period had higher median values and greater variability compared to the “Post-COVID” period, where the values exhibited a tighter spread with an outlier.

The difference in the confidence intervals (CIs) between the “Pre-COVID” and “Post-COVID” periods can be attributed to the significant changes in influenza testing practices and the overall influenza activity during the pandemic. In the post-pandemic period, many countries experienced heightened COVID-19 testing, which often overshadowed influenza testing, leading to reduced influenza testing rates and, in some cases, underreporting of influenza cases. Consequently, the lower CIs observed for the “Post-COVID” period reflect the reduced detection and reporting due to the prioritization of COVID-19 testing. This disparity highlights the challenges in accurately interpreting influenza trends during the pandemic, where changes in testing capacity and public health priorities may have influenced the observed data.

#### Seasonality of influenza analysis.

**Fig 2:**
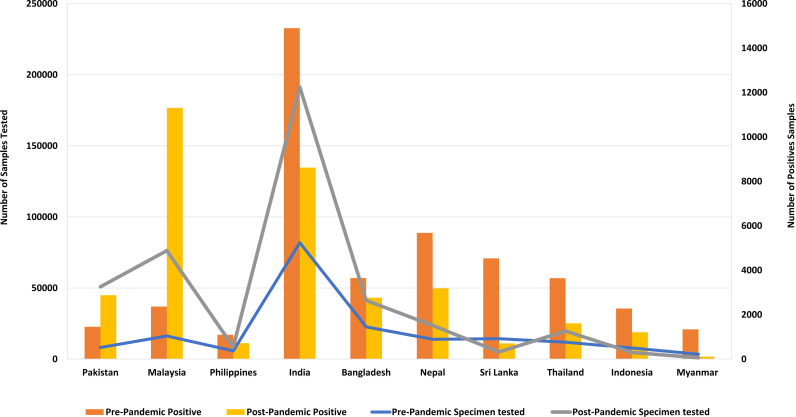
Samples Tested and Positive Samples During Pre- and Post-Pandemic Periods.

Influenza activity for each country was analyzed, by considering the percentage of processed samples that yielded positive results within the concerned time period, as shown in (Fig 2). The countries included in our analysis were categorized into two groups based on their geographical location within the Southern or Northern hemisphere. Specifically, Indonesia was the sole representative from the Southern hemisphere, while the remaining nine countries were situated in the Northern hemisphere of Asia. These geographical boundaries spanned from a latitude of 77° to a longitude of 195°.

Influenza seasons in the Northern hemisphere typically occur between December and March each year. To estimate the duration of each influenza season, we applied the Average Annual Percentage (AAP) technique. This technique allowed us to make predictions regarding the length and timing of influenza seasons within the selected countries, providing valuable insights into the temporal dynamics of influenza activity in both hemispheres [20].


$$\text{AA}{\text{P}}_{\text{i}}\text{= }{\text{n}}_{\text{i}}\text{/}\sum {\text{12}}_{\text{1}}{\text{n}}_{\text{i}}$$

Here, i = month, n = number of cases.

### Ethics declarations

Ethics approval and consent to participate.

Our study was conducted using publicly available data from FluNet, database managed by the World Health Organization (WHO) Global Influenza Surveillance and Response System (GISRS). There was no direct involvement of participants in study design, recruitment or conduct of the study.

## Results

Comparative analysis for influenza activity in South and southeast Asian regions shows an elevated influenza activity in Southern Asia with highest value reaching 878 for influenza A in year 2023. Influenza A[H1] and B (Yamagat) were rarely reported in Southeast Asia and influenza B was rarely reported in South Asia.

### Changes in influenza activity from 2017 to 2023

**Fig 3:**
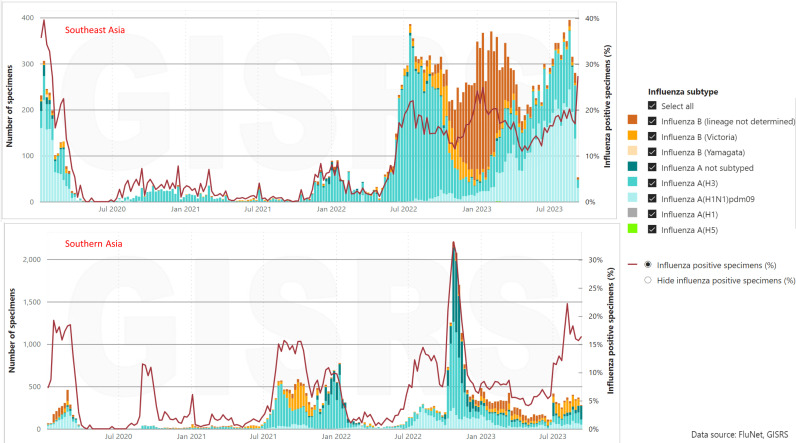
Seasonal Influenza activity from 2020-2023 in Southeast Asia and Southern Asia.

We obtained country-specific data on influenza activity, the number of specimens processed, and the positivity percentage from FluNet for both the pre-pandemic and post-pandemic periods (Table 1). Our analysis covered a total of 10 countries, with 7 located in the WHO SEAR region, 1 in the EMR region, and 2 in the WPR region. Across these countries, we recorded a total of 74,034 cases of influenza positivity from 2017 to 2023. (Fig 3) illustrates the influenza activity in the South and Southeast Asian region from 2020 to 2023. In the first 13 weeks of 2020, prior to the implementation of COVID-19 restrictions, influenza detection rates were comparable to those seen in past years. However, following the introduction of local restrictions in Week 14, influenza activity sharply declined and remained at minimal levels throughout the winter period (Weeks 23–35), even after the relaxation of COVID-19 restrictions. Importantly, the higher testing rates in 2020 suggest that the reduced influenza detection rates were not simply due to decreased healthcare-seeking behavior or fewer tests, which had been initially hypothesized to explain the decline in influenza circulation. This analysis does not include hospital admission data, but it’s worth noting that, unlike COVID-19, which has a lower disease burden in children compared to adults, influenza remains a significant cause of morbidity in children, occasionally leading to hospital admissions on par with those seen in high-risk adults.

**Fig 4:**
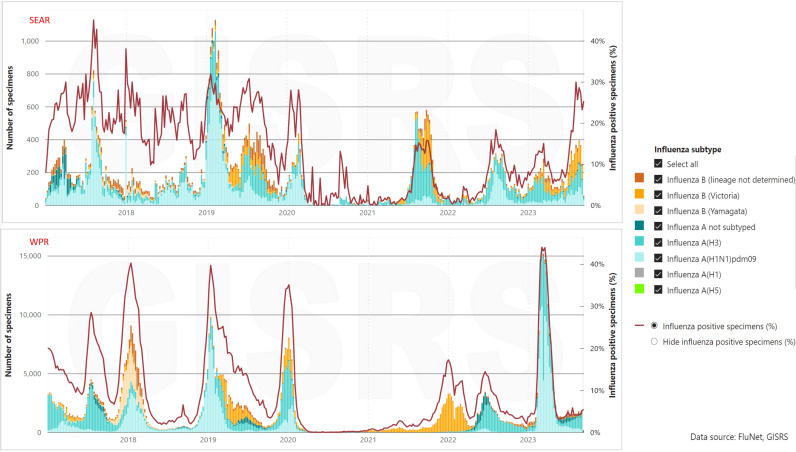
Seasonal Influenza activity from 2017-2023 in SEAR and WPR region.

Significant seasonal fluctuations in influenza activity are evident in the Southeast Asia Region (SEAR), Western Pacific Region (WPR), and Eastern Mediterranean Region (EMR), as depicted in (Fig 4). The study encompassed newly reported cases of COVID-19 across all three WHO regions from 2020 to 2023 (Table 2).

**Table 2:** Comparison of ten Asian countries with pre and post pandemic influenza activity sources through Flunet.

WHO region	Country	Specimen received 2017–2019 (per/1000 population)	Specimen tested	Influenza positive specimen	Specimen received 2020–23/ 1000 population)	Specimen tested	Influenza positive specimen	Total population
EMR	Pakistan	0.0340062	8178	1454	0.21234411	50832	2876	**240,485,658**
WPR	Malaysia	0.4813381	16232	2364	2.2513938	76332	11309	**34,308,525**
	Philippines	0.0493023	5779	1096	0.0729435	8559	714	**117,337,368**
SEAR	India	0.057922	81896	14898	0.1339243	191346	8621	**1,428,627,663**
	Bangladesh	0.00013777	22667	3651	0.2732745	41266	2761	**172,954,319**
	Nepal	0.4594682	13940	5683	0.7664924	23682	3184	**30,896,590**
	Sri Lanka	0.1744804	14326	4532	0.1714201	5069	706	**21,893,579**
	Thailand	0.1654706	11881	3641	0.2744937	19711	1609	**71,801,279**
	Indonesia	0.0281191	7804	2277	0.0168844	4675	1207	**277,534,122**
	Myanmar	0.00006368	3464	1339	0.0000170467	927	112	**54,380,000**

In 2020, a notable decrease in influenza activity was noted in the WPR region, where COVID-19 was initially detected and reported to the WHO. After the implementation of local restrictions in Week 14, influenza activity declined and stayed at low levels throughout the winter period (Weeks 23–35) compared to previous seasons, even after the easing of COVID-19 restrictions.

Moving into 2021, influenza activity remained consistently low, staying below 10% in both the SEAR and WPR regions. However, by 2022, a marked increase in influenza activity was observed in both the SEAR and WPR regions, with the SEAR region registering levels exceeding 70%. Despite this rise, influenza activity in the WPR region remained lower than pre-COVID-19 levels.

Notably, the occurrence of COVID-19 cases declined by January 2023, COVID-19 cases began to decline in the WPR region, which coincided with a notable resurgence of influenza during the spring of that year. The resurgence saw influenza levels climb to as high as 15%. These findings suggest a recovery of influenza activity to pre-pandemic levels, particularly in regions where COVID-19 cases had decreased.

The period from 2022 to 2023 witnessed a return of influenza activity to levels comparable to those observed in 2017 and 2018, signaling a recovery to patterns observed before the pandemic.

Furthermore, an analysis of the duration of influenza epidemics, conducted using the AAP method, indicated that prior to the COVID-19 pandemic (2017–2019), influenza outbreaks typically occurred between July and January, aligning with the seasonal patterns commonly observed in those years.

However, there was a noticeable change in the pattern in July 2021 when the influenza season commenced at a lower intensity. Subsequently, it saw a resurgence in January 2022, as depicted in (Figs 2 and 3).

This shift in the timing of influenza epidemics in the post-COVID period appears to vary across regions, possibly influenced by hemispheric differences.

## Discussion

In this study, we describe patterns of influenza activity and its variations along with subtypes from 2017–2023 with focus on Asian region. Influenza A (H1N1) has always been dominant in both pre and post COVID period in all WHO regions. During the pandemic influenza activity was lowered in all countries in 2020–2021 when COVID cases were reported at peak. Rare influenza B yamagata cases were reported during pandemic. Southeast Asian Region and West Pacific Regions showed a variety in influenza duration as WPR regions first reported COVID cases in 2020. As given the changes of influenza epidemics after COVID, influenza vaccination needs to be introduced to increase immunity for any outbreak [21].

Many previous studies already predicted a rise in infection burden due to influenza in 2022–2023 [22]. The study estimated a recovered rate of influenza in future seasons. Therefore, the impact on next seasons will depend on the duration impact and recovered proportion of influenza post COVID period. Previous studies have estimated a rise in future influenza outbreaks with reference to 2017–2019 influenza rate.

Early research carried out estimated a reduction of 60% in infection rate in US within a ten weeks’ time period. Many non-pharmaceutical interventions as well as vaccines played role to bring influenza to base level during 2020 [23,24]. The occurrence of influenza and COVID lead up to 25% reduction in influenza rate during winter season. Many study models predicted a 1–4-fold rise in influenza epidemic in 2023. So, lack of exposure to influenza during 2020 can lead to weak immunity in upcoming seasons. A study analyzed negligible antibody production against influenza in 2020 [25]. Analyzing previous influenza trends with post COVID trends will help predict future seasonal timings. But these oscillations cannot be accurately measured due to lack of data in post-pandemic period. In our observed countries and their data, winter of 2022 and January of 2023 had a higher positive percentage for influenza cases comparable to previous years. We had a limitation of post COVID time period due to limited data but the available data can show the increased trend of influenza activity.

Prevalence of influenza in Pakistan is 24% and mostly cases are reported from Punjab. Pakistan had dominant influenza season in August-October [26]. There is wide variation both between countries with their data submitted, however, few years’ data from each country was unreported, different countries had different testing and source of data submitted to Flunet.

October 2022 had decreased reporting for sentinel cases of COVID, while influenza kept increasing in Southeast Asia with influenza A subtypes being predominant. In September-October 2022, WHO GISRS laboratories tested more than 133 934 specimens out of which 5323 were tested positive for influenza [27].

The predictive models used in the review were based on the influenza and influenza subtypes for each country, which was not uniform across the countries which might be due to different data collection methods in COVID-19 pandemic during 2020 and 2021. Thus, country specific data for Asian regions was not retrieved. This could have provided more insight into future trends. Second, our model determines the effect of COVID-19 and increased susceptibility to influenza in upcoming seasons due to antigenic changes. Our study demonstrated the absence of seasonal influenza during COVID-19 pandemic which provides evidence for previous studies claiming lowered immunity in individuals post COVID-19 pandemic. This will also affect the prediction models for the onset of seasonal influenza. Moreover, this study didn’t explore the effect the of COVID-19 vaccination on influenza, which also a factor for influenza burden. Our study shows that prediction of peak timing for seasonal influenza before time is important, because temperate regions are expected to experience influenza peaks effected by environmental and immunity factors. Even though influenza B spreads round the year in most tropical regions of southern and south-east Asia, the most effective time for influenza vaccination according to most recent WHO recommended vaccine would be before the monsoon season aiming for protection against influenza A and B peaks. Our finding recommends to take proactive measures to mitigate the health burden.

## Conclusion

This retrospective study highlights a significant drop in influenza cases during 2021 followed by a sudden increase in 2022–2023, indicating a potential future pattern of oscillation between influenza and COVID-like viral diseases. These trends can serve as early indicators during influenza seasons, offering valuable insights for public health regulatory authorities to proactively design programs aimed at increasing influenza immunization rates.

While it’s important to note that our data may not fully represent the disease burden post-pandemic due to limited reporting from some countries to the WHO, it provides a comprehensive view of the present situation and its potential consequences. Past studies have often underestimated the true influenza burden, primarily relying on data from the WHO with limited reporting entities. In contrast, our study provides a quantitative assessment of influenza variation using both monthly and annual data, which can serve as a valuable tool for guiding surveillance and intervention strategies.

In conclusion, our review-based analysis alerts us to the fluctuations in influenza burdenin upcoming seasons and suggests the need for continuous surveillance to monitor potential future trends. Implementing influenza vaccination programs and promoting vaccination among both children and adults can play a crucial role in mitigating the dual burden of influenza in the post-COVID period.

## Limitations

It’s important to acknowledge the limitations of our cross-sectional assessment. Firstly, the reliability of our findings may be affected by the availability of data, as certain WHO regions lacked data for specific influenza subtypes such as AH5 and subtype B. Additionally, there were data gaps for many countries during the COVID-19 season, which could impact the comprehensiveness of our analysis.

Furthermore, we encountered limitations related to the absence of seasonal charts for the Southeast Asian regions, including Pakistan, spanning the period from 2019 to 2023. Lastly, it’s essential to recognize that our study focused exclusively on reviewing influenza activity within Asian countries, which may not provide a complete global perspective on the subject. Some countries (e.g., Malaysia) showed very high increases in testing and positive cases, which could indicate better reporting or a rebound in influenza activity. On the other hand, countries like Myanmar show very low testing rates even in the post-COVID period, which may reflect persistent underreporting or testing constraints.
